# Oral Phyto-thymol ameliorates the stress induced IBS symptoms

**DOI:** 10.1038/s41598-020-70420-4

**Published:** 2020-08-17

**Authors:** Selvaraj Subramaniyam, Shuyou Yang, Bakary N’tji Diallo, Xu Fanshu, Luo Lei, Chong Li, Özlem Tastan Bishop, Sanjib Bhattacharyya

**Affiliations:** 1 Department of Pharmaceutical Science and Chinese Traditional Medicine, Southwest University, Beibei, Chongqing, 400715 China; 2 Research Unit in Bioinformatics (RUBi), Department of Biochemistry and Microbiology, Rhodes University, P.O. Box 94, Grahamstown, 6140 South Africa

**Keywords:** Receptor pharmacology, Biophysical chemistry

## Abstract

Physical stressors play a crucial role in the progression of irritable bowel syndrome (IBS). Here we report a heterogeneous physical stress induced IBS rat model which shows depression and subsequent modulation of IBS by oral treatment of thymol. Oral administration of Thymol reduces the stress induced IBS significantly altering the stress induced gastrointestinal hypermotility, prolonged the whole gut transit time, and increased abdominal withdrawal reflex suggesting gastrointestinal hypermotility and visceral discomfort caused the onset of depression. Immunohistochemical analysis in small intestine and colon of rats shows the decreased 5-HT_3A_R expression level while thymol treatment normalized the 5-HT_3A_R expression in the stressed rats. Molecular docking studies showed that thymol competes with endogenous serotonin and an antagonist, Tropisetron and all have similar binding energies to 5-HT_3A_R. Molecular dynamics simulations revealed that thymol and tropisetron might have similar effects on 5-HT_3A_R. Our study suggest that thymol improves IBS symptoms through 5-HT_3A_R, could be useful for the treatment of IBS.

## Introduction

Stress remains an inextricable part of our life throughout the history of civilization, and perhaps changed its course during the modern era in terms of urbanization and lifestyle. Causes and circumstances of stress could vary in different instances, subsequently changing the manifestations of the cause–effect relationship. Stress in life comes from various origins, such as physical trauma, early life events, loss of parents, physical/sexual abuse, and acts as predisposing risk factors for the development of irritable bowel syndrome (IBS), a functional gastrointestinal disorder (FGID). Physical stressors can alter the gut brain axis affecting the visceral events^1^. Traumatic events can induce changes in the brain sensory response that modulates the neuroendocrine hypothalamus–pituitary–adrenal (HPA) crosstalk^1–4^. A “fight” response generated due to threat (stressor) activates a feedback mechanism to quench the stress to reinstate the system allostasis^2,5^. However a prolonged stressor can ruin the adaptive system to achieve stress homeostasis, and could subsequently turn into pathogenesis of whole body disorders including gastrointestinal tract (GI) of viscera^6,7^. The consequence of stress episodes and associated anxiety is often compensated in adults at the cost of irritable bowel syndrome (IBS)^4^. Hence social stress and relevant maladaptation of life style are often buffered at the expense of IBS. IBS is a complex, polygenic disorder that often includes various symptoms such as abdominal pain and discomfort, visceral hyperalgesia, altered fecal output and GI transit time^8^. Visceral pain can arise from wide arrays of disorders such as gallstone, pancreatitis, esophageal reflux and many others^9^. Nociceptive pain stems from the central nervous system (CNS) innervating viscera to the site of signal transmission^10^. The outcome of visceral pain management has remained unsatisfactory during the last decades including a cost burden of diminished quality of life. However, efforts are ongoing with opioid receptor agonist/antagonist, serotonergic agent, bile acid regulator, which have shown promising results in clinical trials^11^. IBS could arise from different scenario of serotonin level giving different phenotypes; such as either diarrhea, or constipation or none of these^1^. This variable spectrum of IBS symptoms is the key foundation for developing various serotonin based agonist and antagonist to treat IBS. Recent serotonin transporter knock out animal model study suggests mimicking some spectrum of humanized IBS^12^.

## Results

Herein we report a physical stressor mediated IBS in rat model that shows alternation of serotonin receptor (5-HT_3_AR) surface presentation in the intestine and colon. We also report that thymol treatment smooths out the IBS symptoms by altering the 5-HT_3_AR level. Thymol, a mono terpenoid phytochemical found in Southeast Asian herbs call Ajwaan, is used as traditional medicine and food component. It is typically used for bowel related complications and digestion problems^13^. Thymol derivatives have a pharmacologic use as a gastrointestinal modulator, but the biochemical mechanisms are largely unknown. Further studies are required to find out whether thymol could hold a putative potential for pain medication by alleviating the visceral pain associated with IBS and other diseases, modulating the opioid receptor.

**Figure 1:**
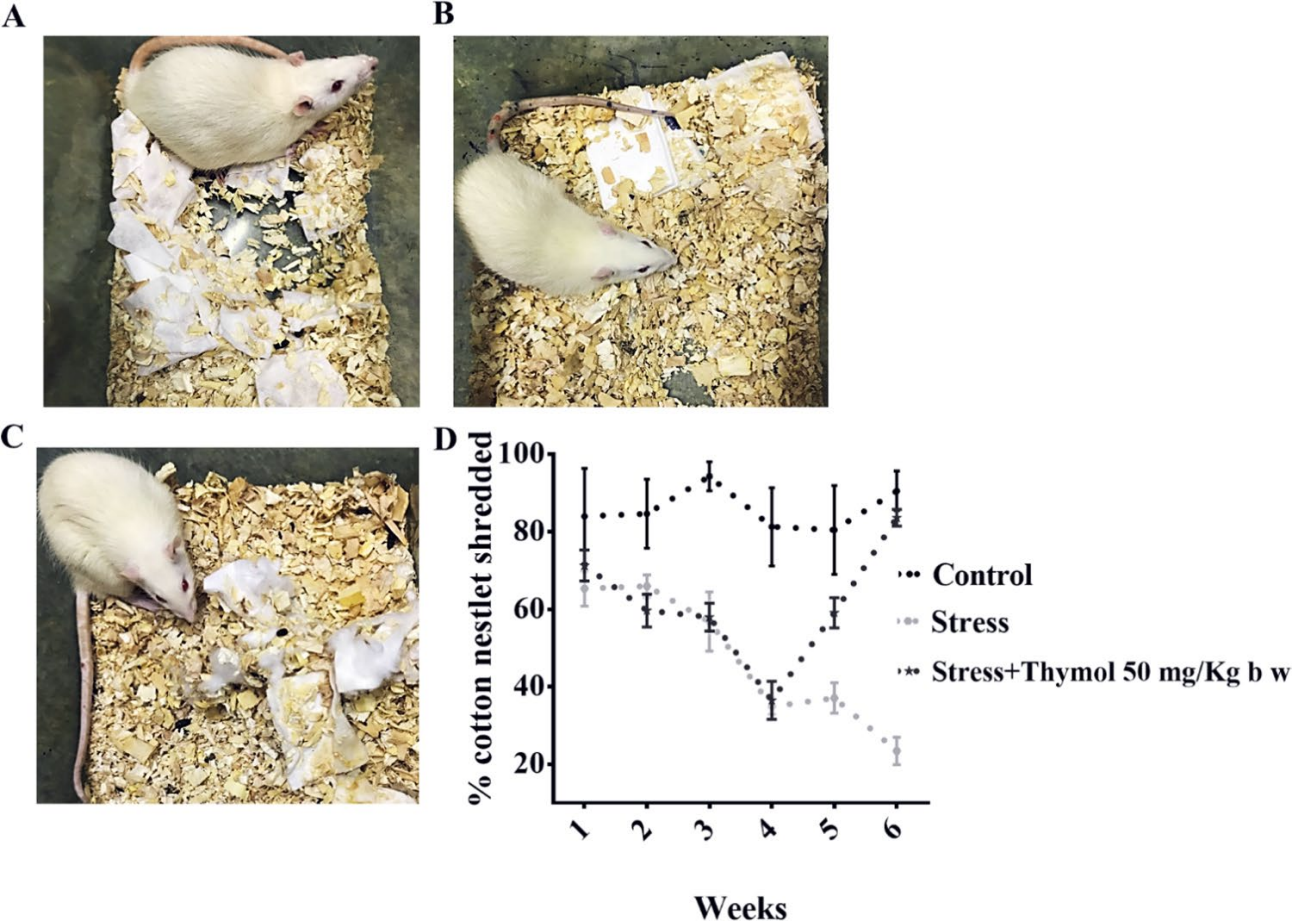
Exposure to chronic stress reveals altered behavioral response in SD rats. Shredding of a cotton nestlet was measured in rat exposed to (**A**) control, (**B**) Stress, (**C**) Stress with treatment of thymol 50 mg/kg b w, and (**D**) Summary data showing that thymol 50 mg/kg b w treated increased the cotton nestlet shredded in chronic stress induced rat. The amount shredded is shown as percentage of total nestlet area. Data shown as mean ± S.E.M (n = 8). *P < 0.05 (one-way ANOVA and TMCT compared with stress).

**Figure 2:**
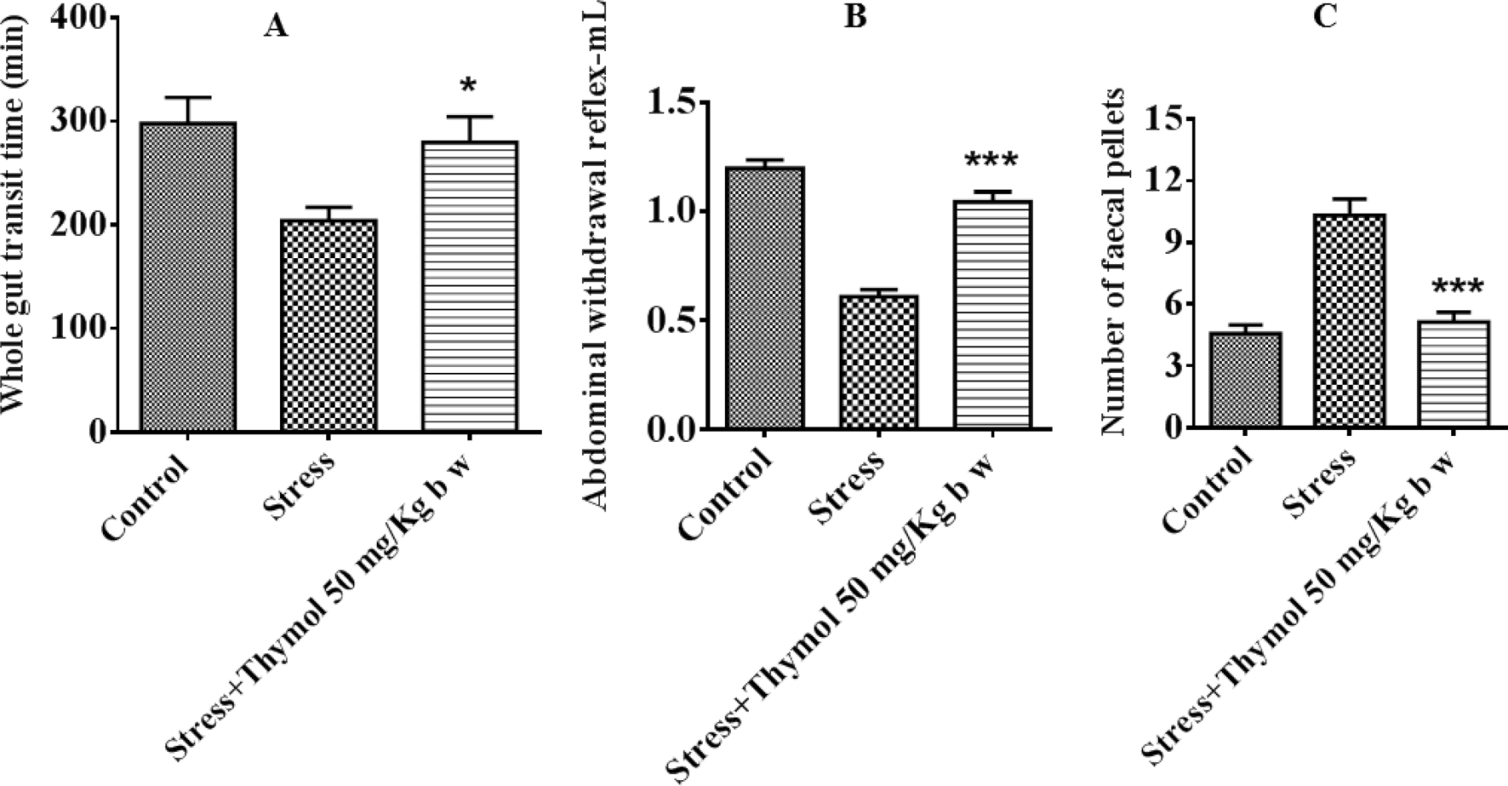
(**A**) Effect of thymol on whole gut transit time (WGTT) in chronic stress induced rat. Summary data shows that thymol 50 mg/kg b w treated prolonged the whole gut transit time in chronic stress induced rat. Data revealed as mean ± SEM (n = 8–10). *P < 0.05 (one-way ANOVA and TMCT compared with stress). (**B**) Effect of thymol on abdominal withdrawal reflex (AWR) in chronic stress induced rat. Summary data showing that thymol 50 mg/kg b w treated increased the abdominal withdrawal reflex in chronic induced rat. Data revealed as mean ± SEM (n = 8–10). ***P < 0.0001 (one-way ANOVA and TMCT compared with stress). (**C**) Effect of thymol on gastrointestinal hypermotility in chronic stress induced rat. Summary data showing that thymol 50 mg/kg b w treated decreased the gastrointestinal hypermotility in chronic stress induced rat. Data revealed as mean ± SEM (n = 8–10). ***P < 0.0001 (one-way ANOVA and TMCT compared with stress).

We used post-natal stress induced rat model of IBS^13^. Rats were given a mixture of heterotypic physical stressor (see “Experimental procedure” section) by alternating modality to mimic the humanized effects in social milieu for a period of 4–6 weeks. Cotton nest behavioral studies were performed preliminarily in order to see whether application of prolonged stress caused their mood fluctuation in terms of depression. We noticed that rat exposed to various stress factors showed visible signs of depression and mood alteration as evident from their participation to play with the cotton nest to reshuffle the cotton (Fig. 1A–C). The tests were scored based on their zeal for participation and performance to play with cotton nest (Fig. 1D). At the end of 4 week of stress, we used gastrointestinal transit (GIT) as readout for both the consequence of stress and subsequent healing ability of thymol (for 2-week treatment starting from week 5 and continued in week 6) to ease out stress induced IBS. GIT is a surrogate marker for evaluating drug efficacy to inhibit bowel abnormalities in IBS, also clinically relevant for the assessment of “organic” disorder such as GI motility^8,14^. Indeed, drugs that target normalizing GIT such as Lubiprostone, Linaclotide turned out to be useful in relieving abdominal discomfort in Phase III trials^1,15^. We found that oral thymol treatment enhanced the GI transit (similar to untreated control animals) which was higher in stress induced animals possibly due to leaky gut (Fig. 2A). This could be due to the stress driven change in local intestinal permeability resulting in modulation of mucosal inflammation and gut sensory visceromotor reflex. Since IBS is often associated with visceral hyperalgesia, fecal pellet output has also been used to study the response of stress and drugs in rodent models; this is in some instances homologous to and in some instances contrary to human GIT studies where the patients as well as healthy control subjects were evaluated in absence of acute stressor^16^. We noticed that fecal output was higher for stress induced rats (Fig. 2B). The thymol treatment significantly decreased the fecal count that was associated with stressed rats (Fig. 2B). This result corroborates other rat model studies that showed stress driven anxiety behavior and evaluation of corticotropin-releasing factor 1 (CRF1), an antagonist altering the accelerated fecal output in human IBS patients as well^16^. Besides leaky gut, stress can change the fecal microbiota that initiated many studies of fecal transplant to donor from IBS patients and subsequent analysis of recipient animal behavior such as visceral hypersensitivity^17^. This archetype is used for the study of anxiety associated psychiatry in mouse model by transferring other’s behaviors. Next, abdominal withdrawal reflex (AWR) was used to measure the visceral hypersensitivity which is pre-clinically and clinically used to assess colorectal distension (CRD)^18^. This method is widely used in human and other rodent subjects as an index of visceral pain for the evaluation of analgesic compounds. We found that the animal under stress showed extreme abdominal discomfort which is evident from the tolerance to external pain stimuli applied by catheterization of balloon in colorectal cavity (Fig. 2C and Fig. S1). The threshold of barostat tolerance was higher for the thymol treated rat upon pain stimuli as evident from the AWRanalysis (Fig. 2C).

**Figure 3:**
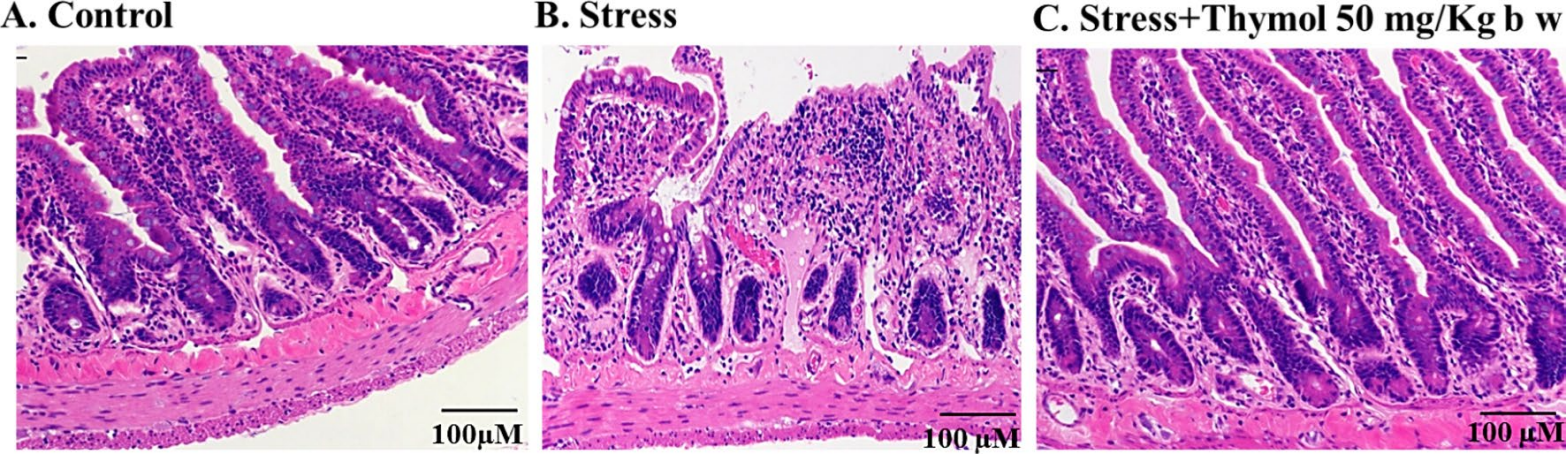
Histopathology of small intestine from chronic stress induced rat. (**A**) Control, (**B**) Stress group: note the crypt flanged with distorted and increasing goblet cells and small intestinal mucosa showing chronic inflammatory changes, epithelial cells of villi damaged and (**C**) Stress induced with treatment of thymol 50 mg/kg b w showing intestinal crypt normal compared to stress.

**Figure 4:**
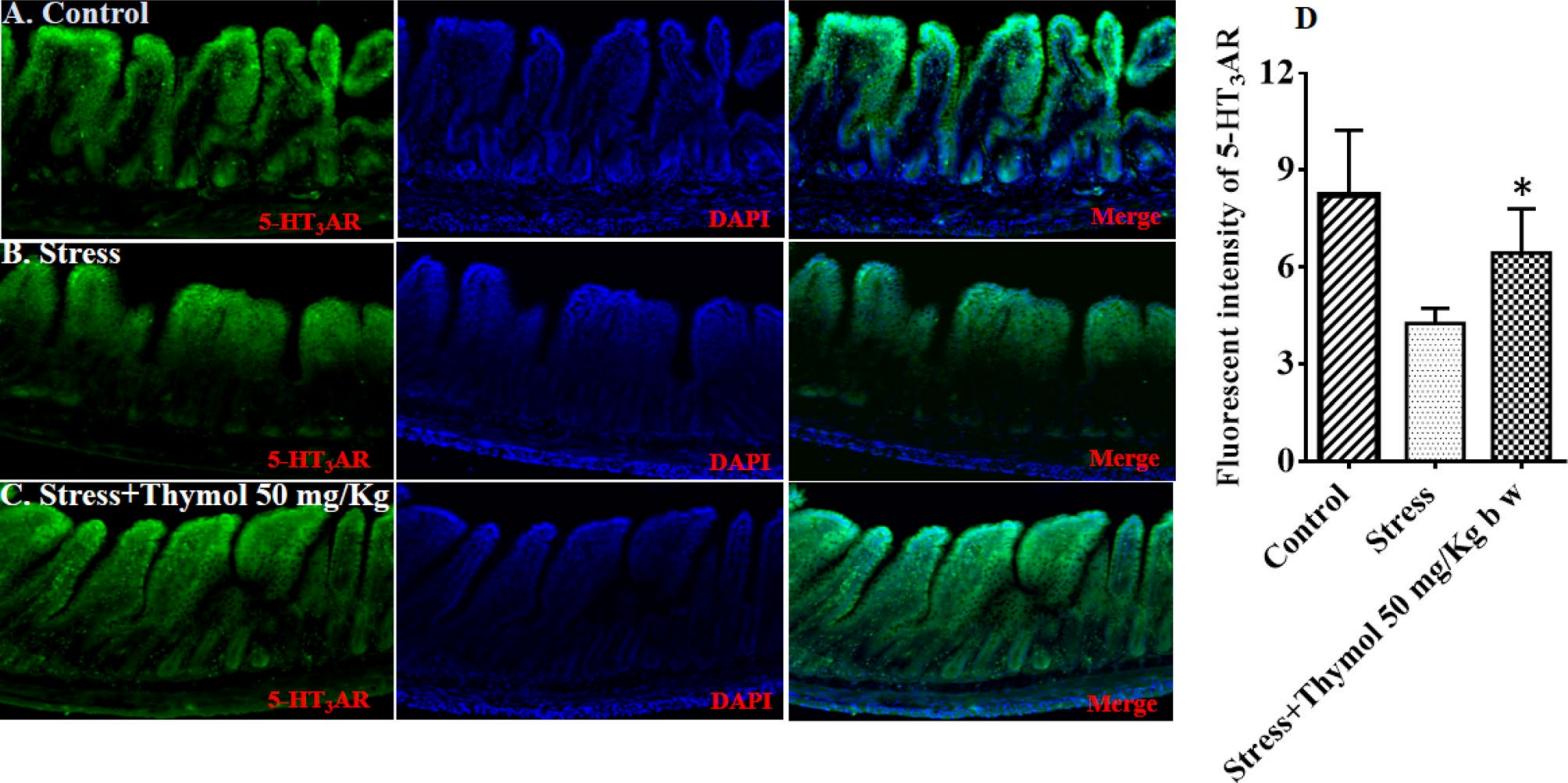
Immunofluorescence detection of 5-HT_3_AR expression in small intestine from chronic stress induced rat. The fluorescent intensity was measured in rat exposed to (**A**) control, (**B**) Stress, (**C**) Stress with treatment of thymol 50 mg/kg b w, and (**D**) Summary data showing that thymol 50 mg/kg b w treated increased the 5-HT_3_AR expression levels in chronic stress induced rat. Data shown as mean ± SEM (n = 8). *P < 0. 05 (one-way ANOVA and TMCT compared with stress).

Next, we wanted to confirm whether the serotonin receptor is the target involved here for the regulation of stress induced IBS and subsequent thymol treatment of rats by oral administration. Serotonin agonist and antagonist are already known to treat IBS related visceral pain^19^. We have chosen 5-HT_3_AR among other serotonin receptor homologues, because 5-HT_3_A antagonist compound is capable of managing stress driven IBS defecation. After the rats were sacrificed, the intestine and colon were used for immunohistochemistry by hematoxylin and eosin staining (IHC), and immunohistofluorescence (IHF) analysis of serotonin receptor. IHC analysis clearly showed that visible symptoms of local intestinal tissue atrophy induced by physical stress, while thymol treatment showed the recovery of the intestine tissue architecture possibly by tissue remodeling (Fig. 3). Similar tissue damage was observed for the colonic tissue (Fig. S2). It was noticed that stress caused reduction of serotonin receptor (5-HT_3_AR) density presence on the surface membrane of the intestine tissue which was enhanced upon thymol treatment (Fig. 4A,B). 5-HT_3_A receptor expression was also upregulated in the case of colonic tissue (Fig. S3). This study indicates that possibly thymol is antagonizing the serotonin receptor to quench the stress mediated IBS that usually results in symptoms of GI hypermotility in rat.

During IBS, opioid receptors are one of the important regulators of pain sensation among the vast array of other receptors involved in pain perception^20,21^. We observed that physical stressor increased the pain sensation as evident from the overexpression of the mu (µ) opioid receptor that jumped higher upon stress induction (Fig. S4). Subsequent thymol treatment of the rats partly adjusts the µ opioid receptor as part of IBS allied visceral pain. However further study is desired to understand how thymol alters the µ opioid receptor axis during pain regulation spurred by IBS. Multi moderators in pain perception are the reason for the complexity to precisely perturb the pain regulator as an IBS related drug target.

**Figure 5:**
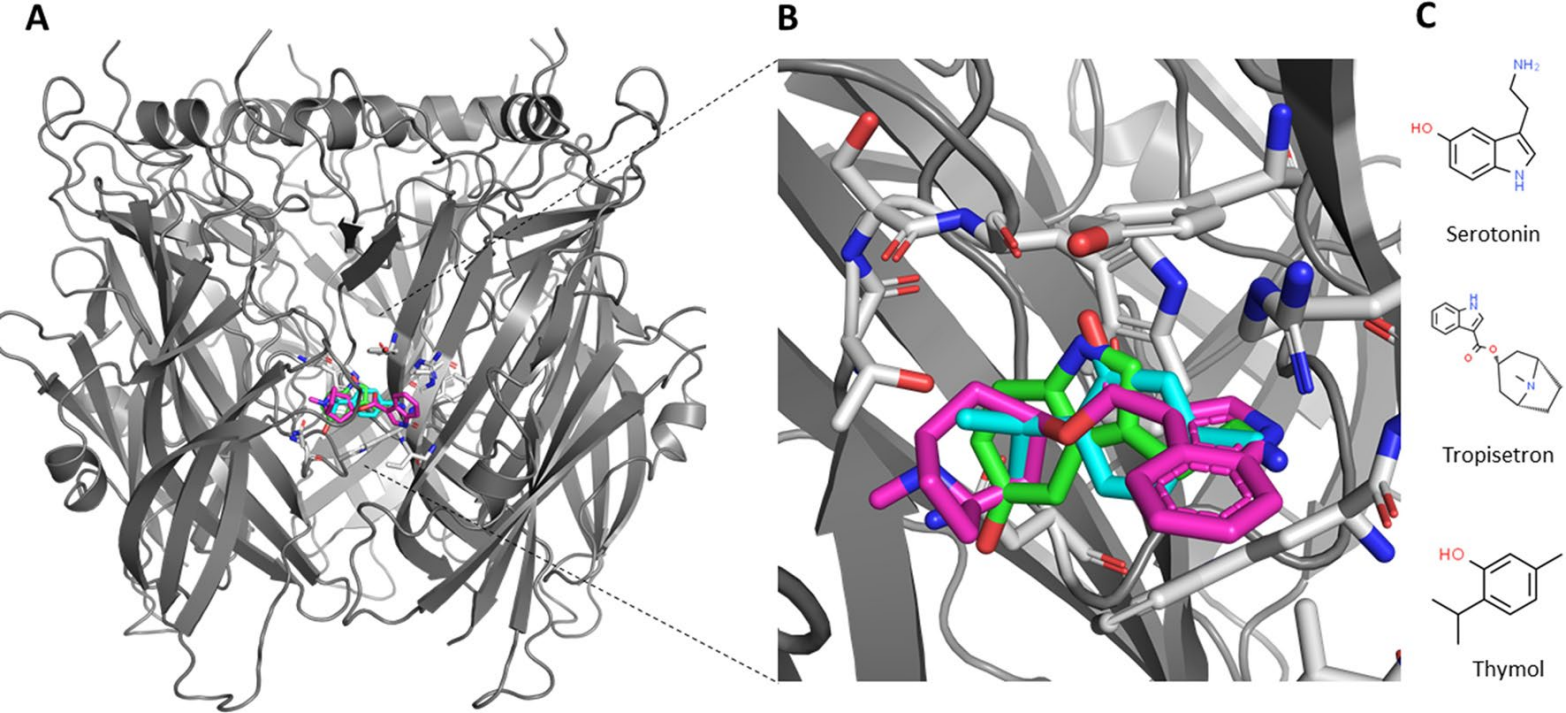
Serotonin and thymol docked receptor. (**A**) Cartoon representation of extra cellular domain (ECD) of serotonin-receptor in transition conformation (PDB ID: 6HIS) with docked serotonin (in green) and thymol (in cyan). Docked compounds and crystalized tropisetron (magenta) are superimposed in active site. (**B**) Zoomed in view of serotonin, Tymol and tropisetron. Serotonin interacting residues are indicated in light grey. (**C**) 2D structural presentation of serotonin, tropisetron and thymol.

A previous study indicated that thymol has a tendency to bind to the transmembrane region of the receptor^22^. Here, we used molecular docking and MD simulation calculations in order to predict whether thymol also has the ability to compete with serotonin towards 5-HT_3_AR that associates with the nociceptive pain signal in IBS via binding to the extracellular domain (ECD). 5-HT_3_A receptor is a pentamer with five equivalent binding sites; the neurotransmitter site is at the subunit interfaces in the ECD^23^. Our molecular docking studies against the entire receptor as well as the different conformations of the ECD of the receptor, as explained in the Experimental Procedure section, clearly revealed that serotonin and thymol bind into the same pocket of the 5-HT_3_AR with similar binding energies (Fig. 5, Fig. S5, Fig. S6 and Table S1, Table S2). Thymol, in all best poses (most energetically favorable), only, bound to the ECD of the structures. All the other poses of thymol also bound to the same domain in all structures except in 6HIS. In that last case, the 7th and 8th poses bound in the intracellular domains in an extreme region close to the extracellular domain. However, the binding region was still different from the one proposed by Lansdell et al.^22^ The very same pocket is also a binding site for tropisetron, an antagonist for 5-HT_3_ receptor, as was shown in crystal structure 6HIS^23^. Further, residue interaction analysis revealed that these compounds interact with protein residues in a similar manner (Table S5, Fig. S7). Interestingly, in 6HIS conformation both tropisetron and thymol interacted with identical residues TRP156 (chain A), TYR207 (chain A), ILE44 (chain E), TRP63 (chain E), ARG65 (chain E); while serotonin differed. However, we also observed similar interacting residues when serotonin binds to all other three conformations (6HIQ, 6HIO, 6HIN). Therefore, the difference in the agonist and antagonist may not be linked to the difference in the compounds’ interacting residues.

To further analyze protein–ligand complexes, MD simulations were performed for the ECD, and root mean square deviation (RMSD), radius of gyration (Rg), root mean square fluctuations (RMSF) and hydrogen bond formation were calculated. RMSD, Rg and hydrogen bond results are presented in Fig. S8. According to RMSD results, all the complexes were stable, and no ligand dissociation was observed. The maximum observed ligand RMSD was ~ 1.25 Å for all ligands except tropisetron in 6HIS which increased up to ~ 2 Å. We also observed that tropisetron moves from its original position that was presented in the crystal structure towards the inner part of the binding pocket (Data not shown). Rg also did not show any significant variation. Concerning hydrogen bonding, serotonin formed a higher number of hydrogen bonds than thymol had in all cases (and also more than tropisetron had in 6HIS). Interestingly, in both 6HIO and 6HIQ, Thymol seemed to rearrange and adopt a more stable pose shown here with its more consistent hydrogen bonding toward the end of the simulation. Thymol in 6HIS presented a special case in which the compound does not make any hydrogen bond, but it is mainly stabilized by hydrophobic contacts.

**Figure 6:**
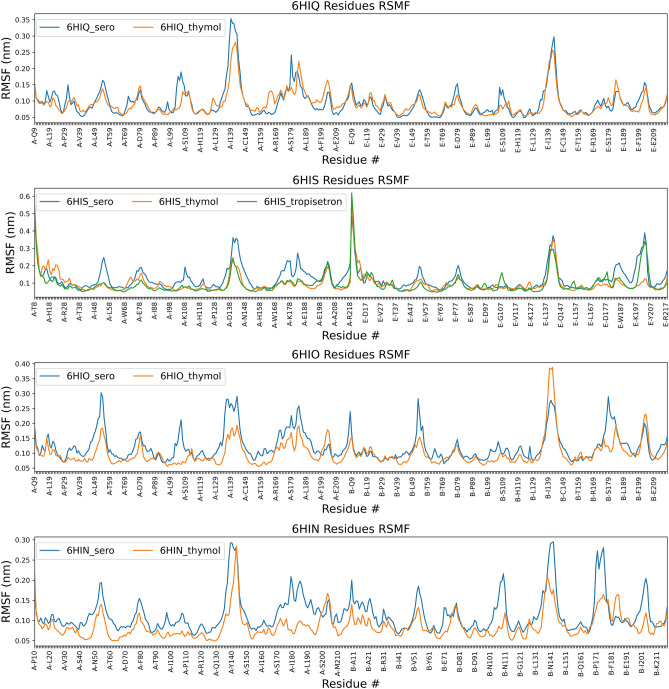
Root mean square fluctuation calculations of extra cellular domain (ECD) of the receptor in compound-bound form for four different conformations. 5-HT_3_A receptor is a pentamer with five equivalent binding sites at the subunit interfaces in the extra cellular domain (ECD). Only RMSF values for residues in chains forming the binding site for the simulated compound are plotted. 6HIQ: intermediate conformation (I2) of 5-HT_3_A receptor with serotonin and TMPPAA (positive allosteric modulator)); 6HIO: intermediate conformation (I1) of the receptor with serotonin; 6HIN: open state of the extracellular domain (ECD) of the receptor with serotonin; 6HIS: transition (T) conformation with tropisetron as antagonist.

Even though, serotonin, thymol and tropisetron are binding to the same binding pocket of the receptor and showing similar residue interaction pattern, RMSF results revealed that the protein is behaving differently in the presence of serotonin and thymol (Fig. 6). In general, residues showed more flexibility when serotonin was bound to protein, contrary to both thymol and tropisetron (6HIS). In the antagonist (tropisetron) bound conformation of the receptor (6HIS), protein residues presented similar behavior in the presence of thymol or tropisetron (Fig. 6). These results might be an indication of antagonist behavior of thymol.

## Discussion/conclusion

**Figure 7:**
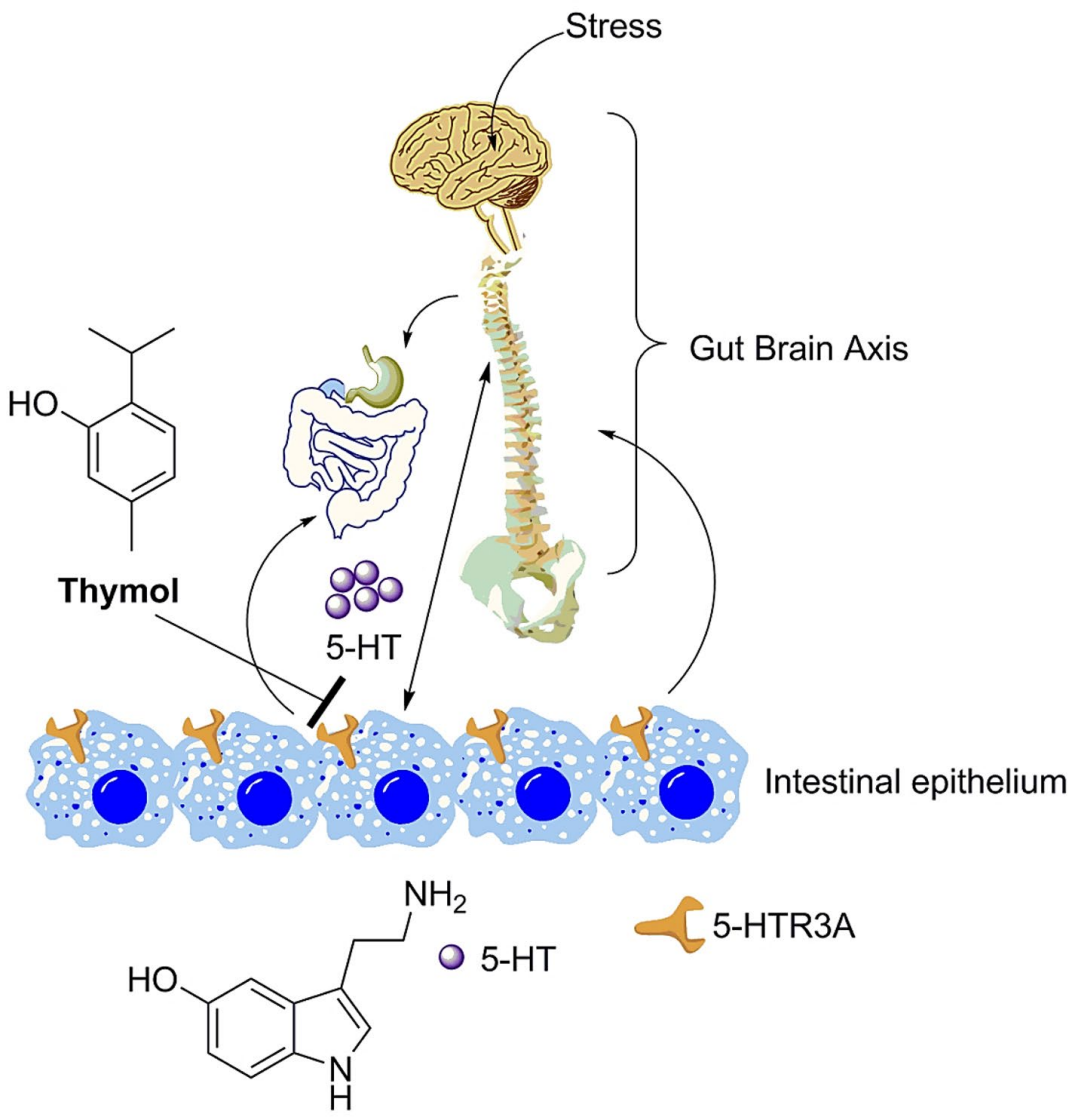
Cartoon presentation of the impact of stress induced IBS during gut brain sensation and subsequent adaptation by producing 5 HT (serotonin). Thymol interferes with the effects of neurotransmitter 5 HT during physical stress mediated IBS and subsequently quench deleterious effects of IBS.

5-HT_3_AR antagonists have been used for IBS treatment modulating the visceral pain situation^19,24^. Thymol has been also used as an essential oil component for various biological benefits^25^. In our study, we find that thymol can manage the intestinal hypermotility and ameliorates the visceral sensitivity that is associated with physical stressor mediated IBS (Fig. 7). Thus, oral thymol administration could be a potential option to handle the leaky gut with subsequent soothing of the IBS symptoms possibly by regulating the serotonin receptor (5-HT_3_AR). Besides animal model, molecular docking simulation study confirms that thymol competes with native ligand towards the same binding site of 5-HT_3_AR. Whether thymol can intervene in the nociceptive pain triggered by noxious stimuli mediating through pain receptor such as opioid and cannabinoid, could certainly lead to a new direction in developing pain medication related to an array of multiple disorders encompassing viscera and innervated spinal cord to GI tract. Thymol mediated recovery of intestinal tissue architecture that was locally damaged by stress induction also requires further evaluation as to whether thymol has the ability of tissue remodeling during IBS mediated tissue loss. Similarly, we could not find conclusive changes from the CaCO-2 cellular competition experiment to obtain more molecular insight for the thymol mediated 5-HT_3_AR antagonism (data not shown). However, 5-HT_3 _R genetic polymorphism (greater C/C genotype) is associated with the severity of IBS patient symptoms, with enhanced anxiety and amygdala^26^. Further investigation is required to understand the precise molecular mechanism of thymol driven 5-HT_3_AR antagonism, even though the 5-HT_3 _R antagonists are viable therapeutic modules to treat IBS and anxiolytic effects.. None the less the non-invasive (oral) validation of thymol as therapeutic target for taming preclinical anxiety associated IBS model in rats warrants further investigation to translate this phytochemical for the application of anxiety associated IBS, other hepatobiliary disease and psycho somatosensory disorders.

Comparing ligand structures, thymol does not present the indole ring present in both serotonin and tropisetron. This moiety is very common among bioactive compounds on the 5-HT_3_A receptor (CHEMBL4972). Indeed, except for its aromatic ring which is common among to 5-HT_3_A receptor antagonists, thymol lacks the carbonyl and the basic amine which are considered as key pharmacophoric point for 5-HT_3_A receptor antagonists. However as previously shown, from histological analysis of 5-HT_3_A receptors, and MD simulation analysis of binding similarity as serotonin and tropisetron, thymol interact with the 5-HT_3_A receptor during stress mediated IBS. These interactions are mainly driven by hydrophobic contacts on its aromatic ring and its substituents as evident from the MD simulation. Hence thymol may present a different scaffold for a new class of 5-HT_3_A modulator and may be worthy of additional structure–activity relation (SAR) study.

## Experimental procedure

### Materials and methods

Thymol and serotonin hydrochloride were purchased from Sigma Aldrich (Catalogue No: G8802B, and H9523), Bradford reagent (Sigma Aldrich, Catalogue No: B6916-500ML), Tris base (Geneview, BT350-500G), RAPA lysis buffer (Beyotime Biotechnology, Catalog No: P0013), Paraformaldehyde (Keshi, Catalog No: 30525-89-4), Sucrose (VWR Life science, Catalog No: M117-500G), PVDF membrane (Millipore, Catalog No: IEVH00005), 5HTR3A rabbit polyclonal and β-actin mouse monoclonal antibody were purchased from Proteintech (Catalog No: 10443-1-AP, and Catalog No: 60008-1-Ig), mu Opioid R rabbit polyclonal (Novus Biologicals, Catalog No: NB100-1620). All methods were performed in accordance with the relevant guidelines and regulation.

### Animals

Adult male Sprague–Dawley (SD) rats at eight weeks old, weighing 222–254 g, were obtained from Chongqing Medical University, Chongqing, China, Animals (n = 30) were transported in a standard cage under similar conditions and were held according to the standards of Animal Ethical Regulations. All methods were performed in accordance with the relevant guidelines and regulation. They were used for the experiment and procured from the animal house of the Southwest University, Chongqing. The animals were maintained in a room with a 12:12 h L:D cycle temperature of 22 ± 2 °C, and Relative humidity of 50–70%. The animals were fed with a balanced commercial diet and water ad libitum. They were allowed for 6-day rest period before the start of the experiment. Experimental protocol approved by International Ethics Committee of Southwest University, Chongqing, China.

### Induction of stress protocol

The stressor was as follows: (a) no water for 12 h, (b) no food for 12 h, (c) wet/dirty bed for 10 min, (d) press tail for 2 min, (e) day and night reverse, (f) shake the cage for 10 min, (g) tight the legs for 3 min. The animals were exposed to one of the seven various restraint stressors for different time manner. The animals exposed to stressor were returned to the animal house after completion following stressor exposure to minimize the disturbance of the control group, group 1. The sequences of the stressors were exchanged alternatively every week. Rats were kept under rest remaining days of the week. After four weeks of stressors the animals were randomly divided into two groups, groups 2 and 3. Each group had eight to ten animals (n = 8–10). Group 3 was treated with 50 mg thymol/kg body weight (Sigma Aldrich, Catalog No: G8802B). Thymol was dissolved in 0.9% NaCl along with tween 80 (1%) and then orally fed on the fifth and sixth consecutive weeks to the animals of group-3 as indicated. The experiments were conducted for six weeks.

### Cotton nestle shredding test

Rats were individually placed in a clean cage with a cotton material comprising 5 cm^2^ squares of compressed cotton allowed in the cage at 12 h; the percentage of the area of cotton shredded was measured by NIH software (ImageJ).

### Whole gut transit time (GIT) test

In the morning at 8 a.m. of each experimental animal were transferred into individual empty polyethylene cage and were left to acclimatize to the cage for 1 h. 5% Evan blue and 5% gum Arabic dissolved in 0.9% saline was feed orally to those rats. Rats were returned to their individual cage. The time from the end of the experiment to the notice of the first blue fecal pellet was measured in minutes and constituted the whole gut transit time. During the whole gut transit time the number of fecal pellets in each cage was counted for gastro intestinal hypermotility.

### Abdominal withdraw reflex (AWR) test

Rats were anaesthetized by ethyl ether and catheter with syringe was inserted (about 8 cm after lubrication with paraffin oil) through anus. When the rats woke up, they were put in a special transparent plastic cage; after the rat adapted to the environment, air was gradually injected into the catheter to expand the intestinal tract, recording the volume of injected air when rats reach to three points. This experiment was repeated three times, with an interval of five minutes.

### Tissue processing for histology and immunofluorescence

#### Intestine and colon for histology

At the end of the behavioral experimental, animals were autopsied after anesthesia with ether. 2 cm of intestine and colon were removed, placed in 4% paraformaldehyde overnight, and sent for histologic processing. The small intestine and colon were fixed in formalin, dehydrated in grade of alcohols and xylene, embedded in paraffin wax, cut into 5 µm cross section by microtome. A cross section of intestine and colon were attached in pre albumin coated slide to be stained with hematoxylin and eosin. Intestine and colon section slides were imaged using a NIKON Eclipse e100.

#### Intestine and colon for immunofluorescence histochemistry

The intestine and colon were dissected and stored in fixative overnight, after that washed with PBS 3–4 times, and transferred into 30% sucrose (VWR Life Science, Catalog No.: M117-500G), stored at 4 °C. Intestine and colon were sectioned at 20 µm cut on a freezing cryostat. Then the sections were rinsed three times with Tribase saline and 0.1% tween 20 (TBST). Non-specific antibody reactions were blocked with 5% horse serum (v/v) in TBST. Sections were incubated with primary 5-HT_3_AR rabbit polyclonal (Proteintech, Catalog No.: 100443-1-AP, dilution 1:200) and mu Opioid R rabbit polyclonal (Novus Biologicals, Catalog No: NB100-1,620, dilution 1:200) in TBST containing 5% horse serum at 4 °C for overnight. After being washed three times in TBST, sections were incubated with secondary anti rabbit IgG (H + L) F(ab′) fragment conjugate with Alexa Fluor 488 antibody (Cell signaling Technology, Catalog No: 4480S, dilution 1:250) at room temperature for 1 h, keeping in a dark humidified chamber. Finally, sections were cover slipped with antifade mounting medium with DAPI (Vectashield, Catalog No: H-1200). Fluorescent images were captured with Leica dmi8, and processed using the program Leica Application Suite X 3.3.3.16958.

### Statistical analysis

All data were analyzed using Prism 6 software (GraphPad software). Experiments were analyzed using one-way ANOVA and Tukey corrections for multiple testing between categories. Data is presented with means ± standard error of the mean (SEM) shown as line and sticker.

### Data retrieval and molecular docking studies

For the entire receptor, which includes intra and extra cellular domains, 4PIR was used^27^. For extra cellular domain (ECD) four receptor structures in different conformations were retrieved from Protein Data Bank (PDB). These are 6HIQ in intermediate conformation (I2) of 5-HT_3_A receptor with serotonin and TMPPAA (positive allosteric modulator); 6HIO in intermediate conformation (I1) of the receptor with serotonin; 6HIN in open state of the extracellular domain (ECD) of the receptor with serotonin; 6HIS in transition (T) conformation with tropisetron as antagonist. Thymol structure was obtained from PubChem (CID: 6989). Missing atoms in 6HIS ECD were modelled using Prime version 5.4 (r012)^27^.

The docking validation was, first, done by re-docking serotonin to the 5-HT_3_ receptor (6HIQ) using blind docking approach via QuickVina-W^28^. The RMSD value between crystalized and docked serotonin was RMSD 2.2 Å (Fig. S9). Blind docking experiments were, then, repeated against full length protein (4PIR) as well as against the ECD domain in four different conformations. Thymol, serotonin, tropisetron and NAG (*N*-acetly-d-Glucosamine, the co-crystal ligand in 4PIR) were docked in the 5 five structures. The complete docking parameters are in Table S3 and Table S4. Root mean square deviation (RMSD) values were computed using GROMACS (Version 5.1.2)^29^ without least-squares fitting of the structures.

### Molecular dynamics simulations and analysis

Nine MD simulations were done using the ECD of the receptor extracted from different conformations. TMPPAA was not included in the simulations. Systems were simulated in a dodecahedron box. The distance between the solute and the box was set to 1.0 nm. TIP3P water model was used with a concentration of 0.15 M Na + (sodium) and Cl− (chloride) ions. Energy minimization was done via steepest descent method with a maximum force set at < 1,000.0 kJ/mol/nm and a maximum number of steps to 50,000. This was followed by equilibration at 300 K and 1 atm with 50 ps of MD simulation in the isothermal-isobaric ensemble and subsequently in the canonical one. A cutoff of 10 Å was used for the Lennard–Jones and short-range electrostatic interactions. The smooth particle mesh Ewald method and a fourth-order interpolation scheme were used for the long-range electrostatic interactions. The leap-frog algorithm was used for integration. Ligand topology files were generated via ACPYPE^30^ using a total charge of zero for all ligands. Simulations were conducted on a remote machine at Center for High Performance Computing (CHPC) with GROMACS version 2016 using the Amber ff99SB-ILDN^31^ force field. The generated trajectories were analyzed with GROMACS modules using RMSD, radius of gyration (Rg) and root mean square fluctuations (RMSF) to assess protein stability. Ligand stability was assessed using the RMSD of the ligand heavy atoms after being least-squares fitted to the protein backbone and also using the number of hydrogen bonds they formed.

## Supplementary information

Supplementary Information.
