# Supplementary material for: Oral Phyto-thymol ameliorates the stress induced IBS symptoms
Source: Sci Rep. 2020 Aug 17;10:13900. doi: 10.1038/s41598-020-70420-4 (PMC7431530; doi:10.1038/s41598-020-70420-4)
Supplement: Supplementary file 1 — Supplementary Information. [file 41598_2020_70420_MOESM1_ESM.docx]

**Title: Oral Phyto-thymol ameliorates the stress induced IBS symptoms**

Selvaraj Subramaniyam,^1*^ Shuyou Yang,^1*^ Bakary N'tji Diallo,^2^ Xu Fanshu,^1^ Luo Lei, Chong Li,^1^ Özlem Tastan Bishop,^2#^ & Sanjib Bhattacharyya ^1#^

^1^Department of Pharmaceutical Science and Chinese Traditional Medicine, Southwest University, Beibei, Chongqing, 40075, China

^2^Research Unit in Bioinformatics (RUBi), Department of Biochemistry and Microbiology, Rhodes University, P.O. Box 94, Grahamstown, 6140, South Africa

#E-mail for correspondence: o.tastanbishop@ru.ac.za and sanjib2017@swu.edu.cn

* Equal contribution

Keywords: Thymol, Serotonin Receptor, Anxiety, IBS Therapy, Molecular Docking, Molecular Dynamics Simulation

**Supplementary Tables:**

**Supplementary table S1**: Best poses binding energies of serotonin, thymol and tropisetron in docking on full structures (Extracellular and Membrane domains).

**Supplementary table S2:** Serotonin and thymol docked extracellular domain binding energies.

**Supplementary table S3:** Full structures (Extracellular and Membrane domains) blind docking parameters

**Supplementary table S4:** Extracellular domains blind docking parameters

**Supplementary table S5:** Receptor-serotonin, -thymol and -tropisetron interacting residues. Interaction types are presented in Supplemental figure S5.

**Supplementary table S6:** Extracellular domain blind docking parameters

**Supplementary table S7:** Serotonin and thymol interacting residues

**Supplementary Figures:**

**Supplemental figure S1.** Visceral pain behavioral evaluation of an image illustration of visually based measure of abdominal withdraw reflex pattern of different of experimental groups. (A) Control, (B) Stress, and (C) Stress with treatment of Thymol 50 mg/Kg b w.

**Supplemental figure S2**. **Histopathology of colon from chronic induced stress of rat.** (A) Control, (B) Stress group note the crypt distorted and decreased goblet cells and colon mucosa showing chronic inflammatory changes, vesicular nuclei prominent damaged, scattered, and (C) Stress induced with treatment of Thymol 50 mg/Kg b w showing that intestinal crypt normal compared to stress.

**Supplemental figure S3. Immunofluorescence detection of 5-HTR3-A expression in Colon from chronic stress induced of rat.** The fluorescent intensity was measured in rat exposed to (A) control, (B) Stress, (C) Stress with treatment of Thymol 50 mg/Kg b w, and (D) Summary data showing that Thymol 50 mg/Kg b w treated elevated the 5-HTR3-A expression levels in chronic stress induced rat. Data shown as mean ± S.E.M (n=2).

**Supplemental figure S4. Immunofluorescence detection of Mu opioid receptor expression in small intestine from chronic stress induced of rat.** The fluorescent intensity was measured in rat exposed to (A) control, (B) Stress, (C) Stress with treatment of Thymol 50 mg/Kg b w, and (D) Summary data showing that Thymol 50 mg/Kg b w treated decreased the 5-HTR3-A expression levels in chronic stress induced rat. Data shown as mean±S.E.M (n=2).

**Supplemental figure S5**. **Thymol, serotonin and tropisetron docked in 4PIR.** (A) Cartoon representation of serotonin-receptor (PDB ID: 4PIR) with docked serotonin (in green), thymol (in cyan) and tropisetron in magenta.

**Supplemental figure S6. Thymol, serotonin and tropisetron docked in 6HIQ.** (A) Cartoon representation of serotonin-receptor (PDB ID: 6HIQ) with docked serotonin (in green), thymol (in cyan) and tropisetron in magenta.

**Supplemental figure S7**. **2D interaction plot of receptor-ligand complexes.** Only ligand poses taken to MD simulation are shown. The 2D plots are obtained from Discovery Studio Visualizer V1.7.2.0.16349.

**Supplemental figure S8. Post molecular dynamics (MD) analysis.** (A) Protein root mean square deviation (RMSD), (B) Protein radius of gyration (Rg), (C) Ligand root mean square deviation (RMSD), (D) Hydrogen bond frequency between protein and ligand. Color code for (A), (B), (C) is given in (A). RMSD and Rg values are presented in nanometer (nm) and time in nanosecond (ns).

**Supplemental figure S9**. **Docking validation.** Crystalized serotonin was re-docked to the structure (green) and compared to the blind docking result (yellow). The RMSD value between crystalized and docked serotonin was RMSD 2.2 Å.

Supplementary table S1:

**Table S3**: Best poses binding energies in docking on full structures (Extracellular and Membrane domains).

| Complexes | Lowest Binding energy (kcal/mol) |
| --- | --- |
| 4pir_NAG | -5.6 |
| 4pir_sero | -6.6 |
| 4pir_thymol | -6.9 |
| 4pir_tropi | -8.8 |
| 6hin_NAG | -6.4 |
| 6hin_sero | -7.8 |
| 6hin_thymol | -7.9 |
| 6hin_tropi | -8.0 |
| 6hio_NAG | -6.5 |
| 6hio_sero | -7.8 |
| 6hio_thymol | -8.2 |
| 6hio_tropi | -8.6 |
| 6hiq_NAG | -6.7 |
| 6hiq_sero | -8.0 |
| 6hiq_thymol | -7.9 |
| 6hiq_tropi | -8.9 |
| 6his_NAG | -5.6 |
| 6his_sero | -6.7 |
| 6his_thymol | -6.5 |
| 6his_tropi | -9.3 |

**Table S4**: Serotonin and thymol docked extracellular domain binding energies

| Proteins | Best pose binding energies (kcal/mol) | | |
| --- | --- | --- | --- |
|  | Serotonin (Crystal) | Serotonin (redocked) | Thymol |
| 6HIQ | - 6.4 | -8.0 | -7.9 |
| 6HIS Tropisetron (Crystal) | - 4.6 Tropisetron (Crystal) | -9.2 | -6.5 |
| 6HIN | - 5.9 | -7.9 | -8.0 |
| 6HIO | -5.8 | -7.8 | -8.2 |

**Table S5**: Full structures (Extracellular and Membrane domains) blind docking parameters

| Receptor | 4PIR | 6HIN | 6HIO | 6HIQ | 6HIS |
| --- | --- | --- | --- | --- | --- |
| center_x | 154.29 | 124.68 | 124.68 | 128.05 | 124.69 |
| center_y | 203.38 | 124.68 | 124.67 | 128.06 | 124.69 |
| center_z | 265.85 | 137.73 | 129.25 | 131.77 | 125.23 |
| size_x | 147.95 | 87.68 | 85.02 | 85.71 | 85.17 |
| size_y | 136.67 | 87.48 | 84.98 | 85.59 | 84.97 |
| size_z | 167.21 | 117.05 | 155.93 | 157.93 | 161.94 |
| exhaustiveness | 6000 | 6000 | 6000 | 6000 | 6000 |
| CPU | 24 | 24 | 24 | 24 | 24 |

**Table S6:** Extracellular domain blind docking parameters

| Receptor | 6HIN | 6HIO | 6HIQ | 6HIS |
| --- | --- | --- | --- | --- |
| center_x | 124.68 | 124.68 | 128.07 | 124.69 |
| center_y | 124.68 | 124.68 | 128.05 | 124.69 |
| center_z | 156.34 | 156.67 | 159.61 | 153.22 |
| size_x | 82.68 | 80.02 | 80.71 | 80.17 |
| size_y | 82.48 | 79.98 | 80.59 | 79.97 |
| size_z | 67.42 | 66.99 | 68.35 | 69.71 |
| exhaustiveness | 529 | 512 | 516 | 513 |
| CPU | 4 | 4 | 4 | 4 |

| Proteins | Interacting residues | |
| --- | --- | --- |
|  | Serotonin (Crystal) | Thymol |
| 6HIQ | A-ILE201  E-ARG65  E-TYR64 | A-TRP156  A-TYR207  A-PHE199  A-ASN101  A-THR154  A-ILE201  E-TYR126  E-TYR64  E-TRP63  E-ARG65  E-ILE44 |
| 6HIS | Serotonin (Docked)  A-TRP156  A-ASN101  E-TRP63  Tropisetron (Crystal)  A-TRP156  A-TYR207  E-ARG65  E-ILE44  E-TRP63 | A-TRP156  A-TRP207  E-ARG65  E-ILE44  E-TRP63  E-TRP126 |
| 6HIN | A-ILE44  A-ARG65  A-TRP63  A-TYR126  A-TYR64  A-LYS127  B-THR154  B-PHE199  B-TYR207  B-ILE201  B-TRP156  B-SER155 | A-TRP63  A-TYR126  A-ILE44  B-TRP156  B-TYR207  B-PHE199 |
| 6HIO | A-ARG65  A-LYS127  A-TYR64  B-TYR207 | A-ARG65  A-TRP63  A-TYR126  A-ILE44  B-TRP156  B-TYR207  B-PHE199 |

**Table S7:** Serotonin and thymol interacting residues

**Supporting Figures S1:**


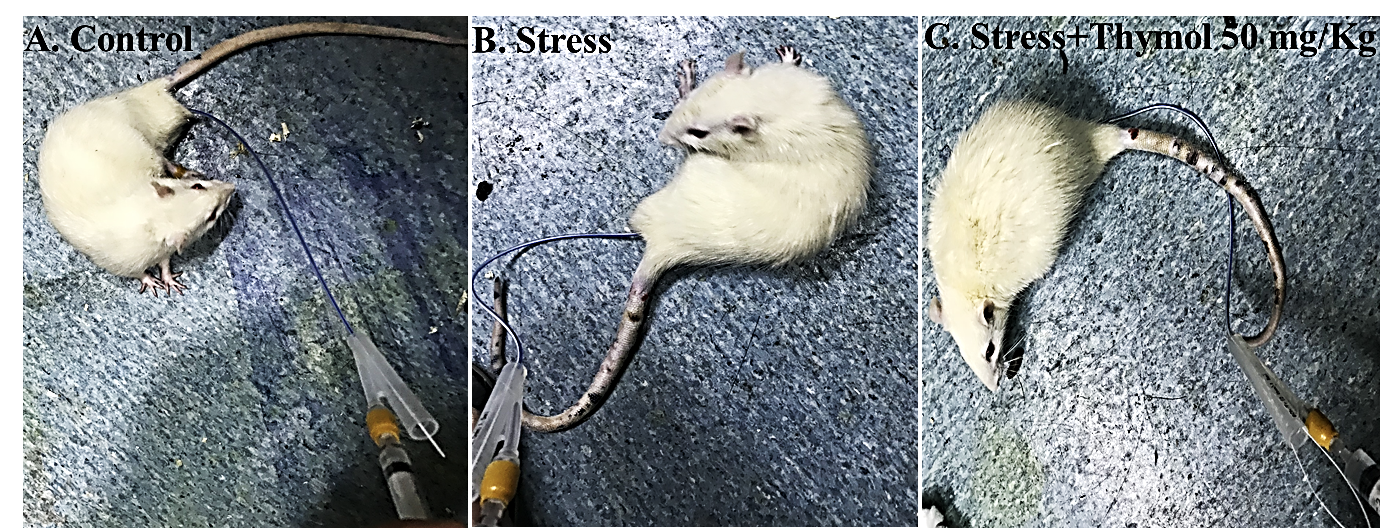


**Supporting Figures S2:**


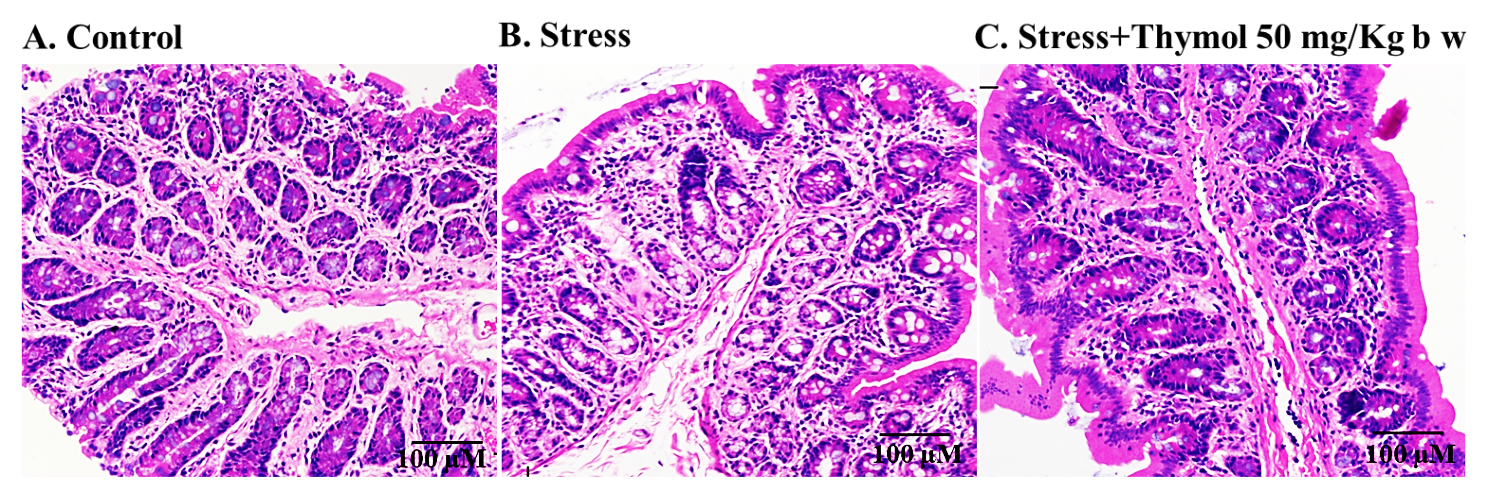


**Supporting Figures S3:**


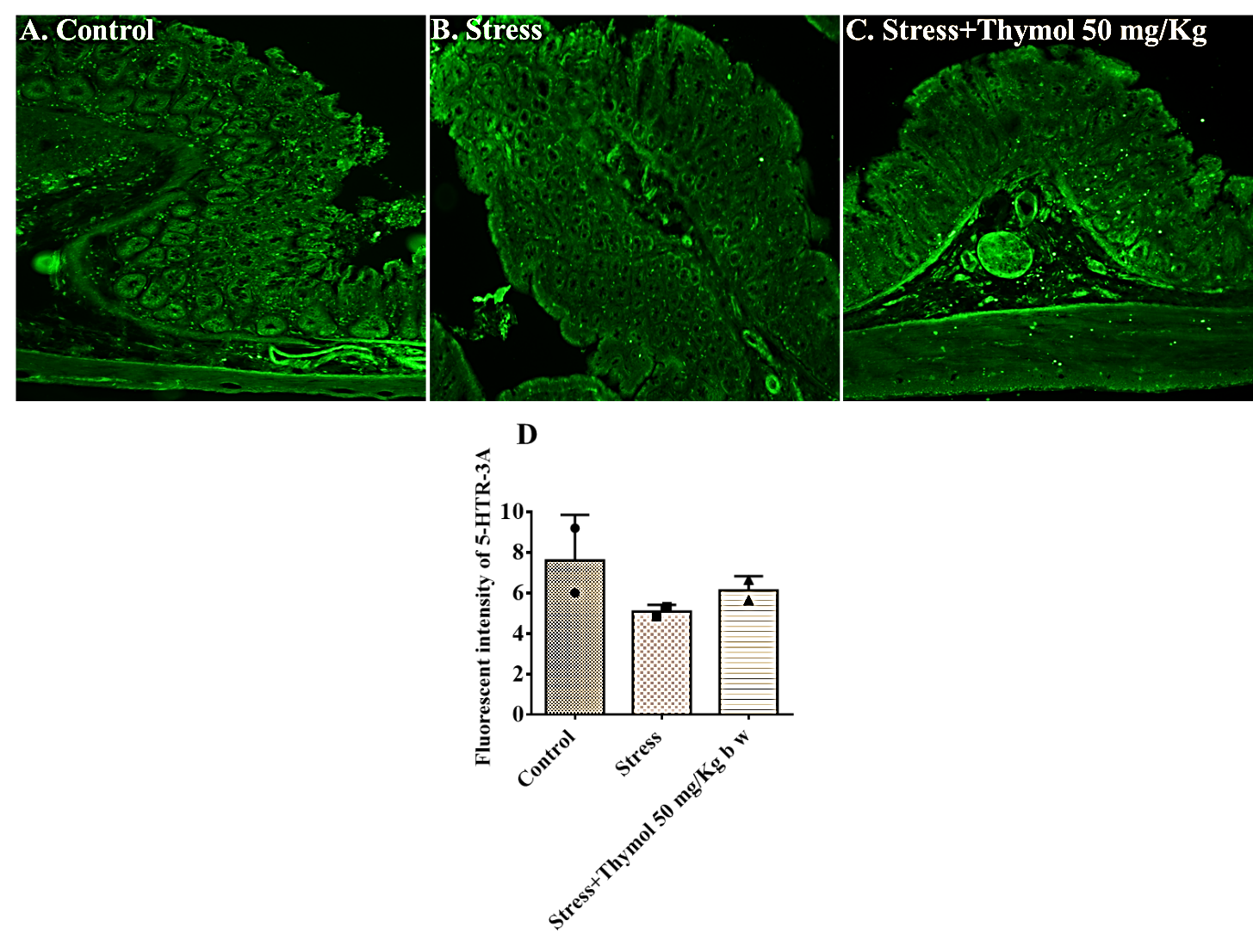


**Supporting Figures S4:**


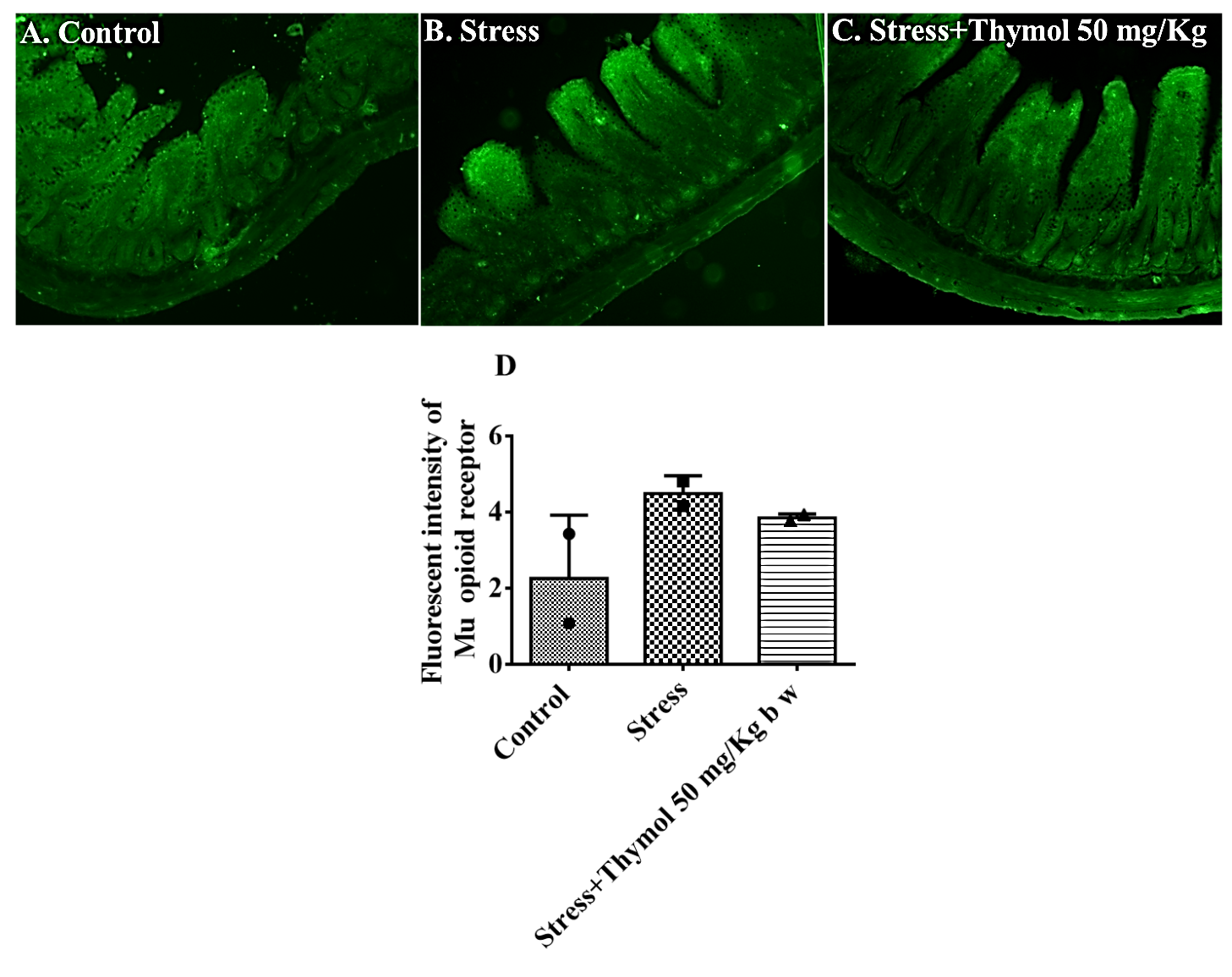


**Supporting Figure S5:**


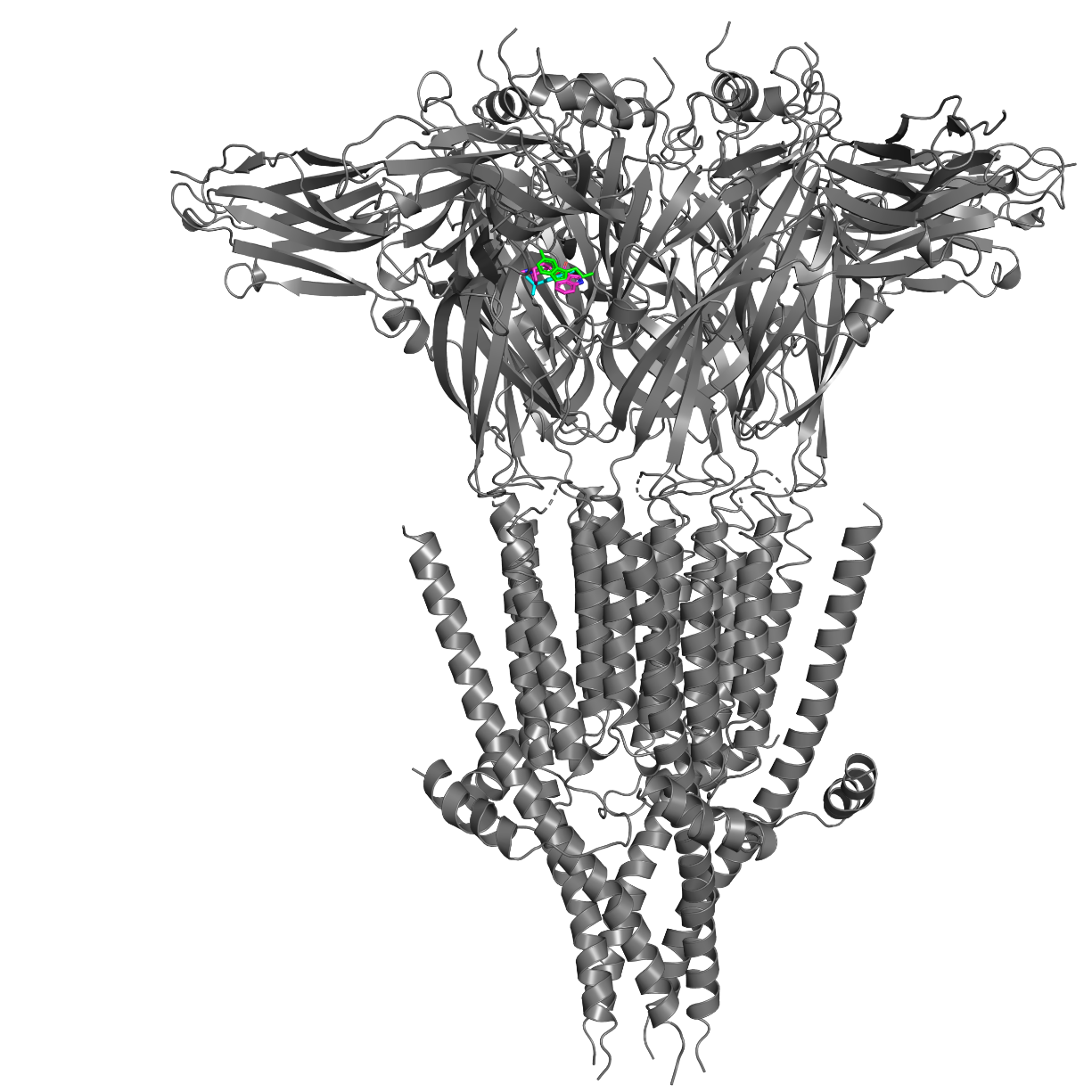


**Supporting Figure S6:**


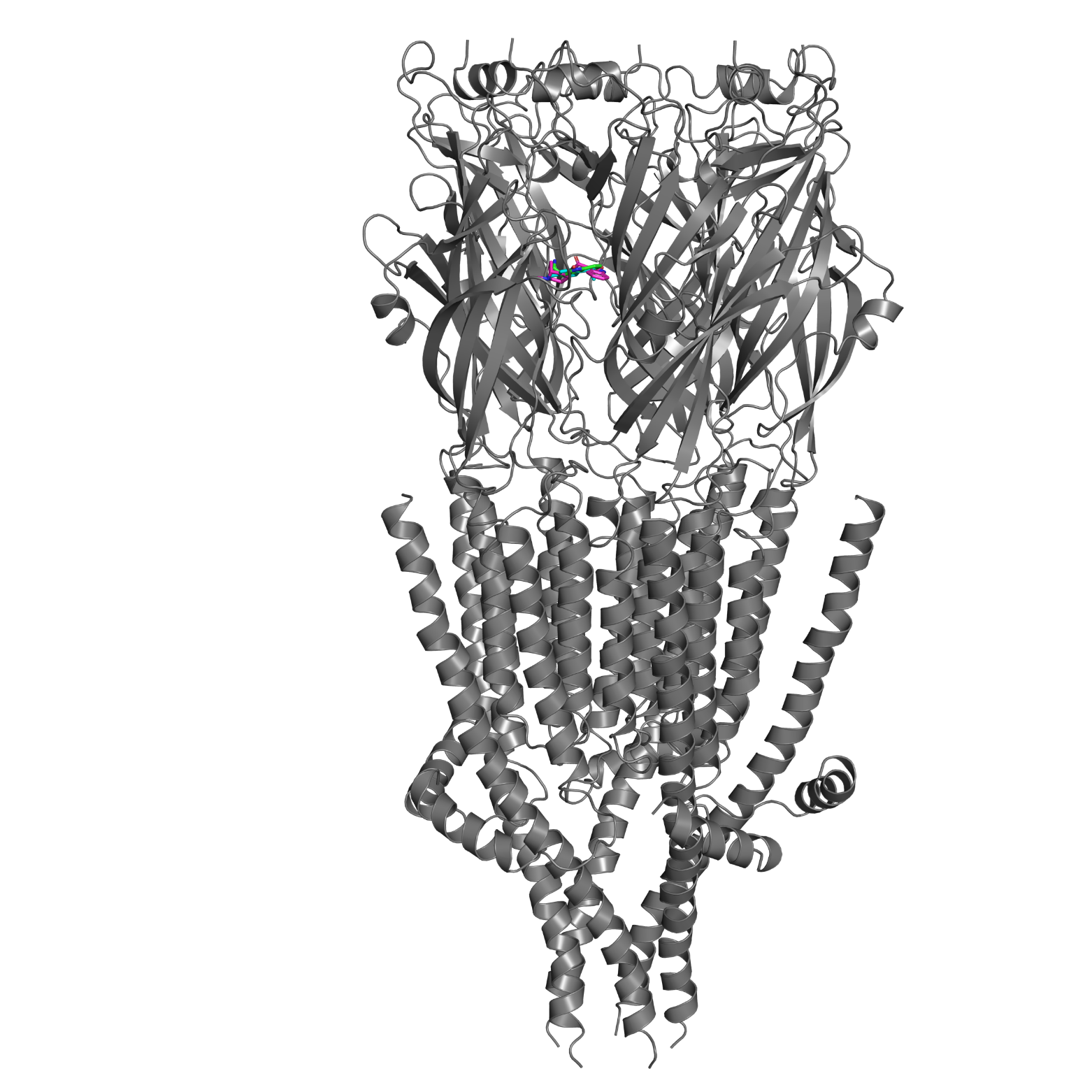


**Supporting Figure S7:**


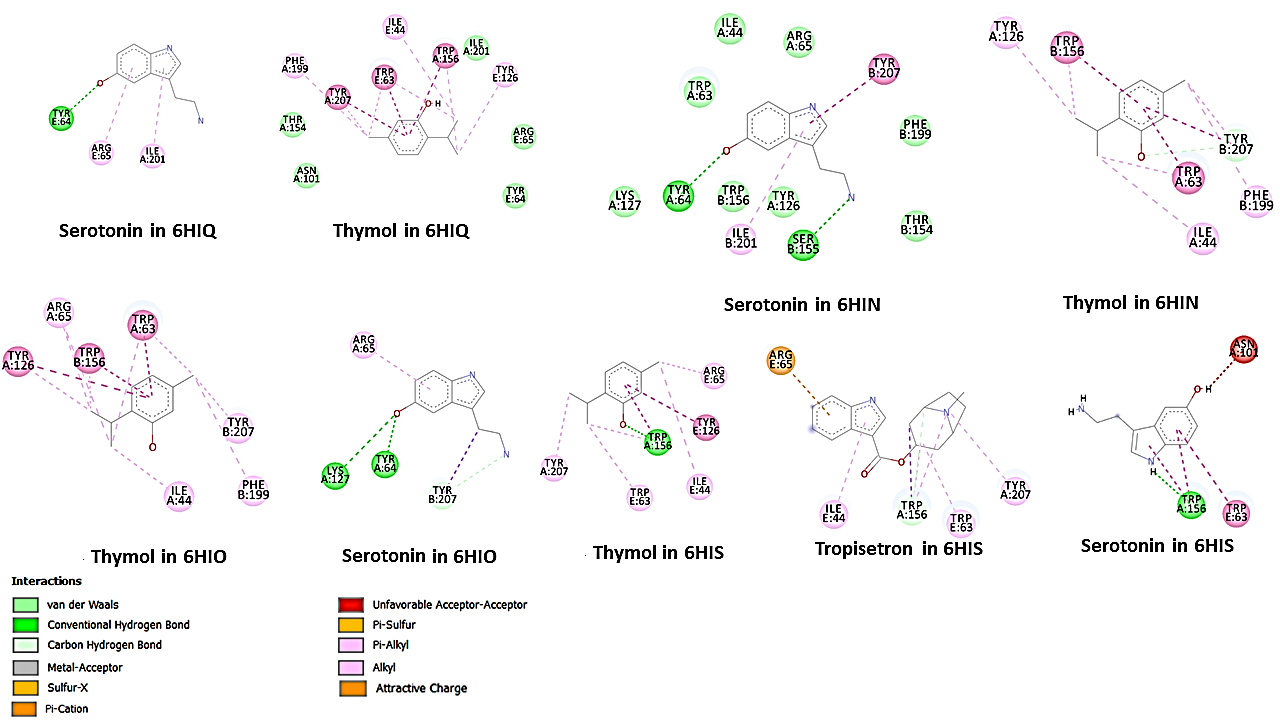


**Supporting Figures S8:**

**
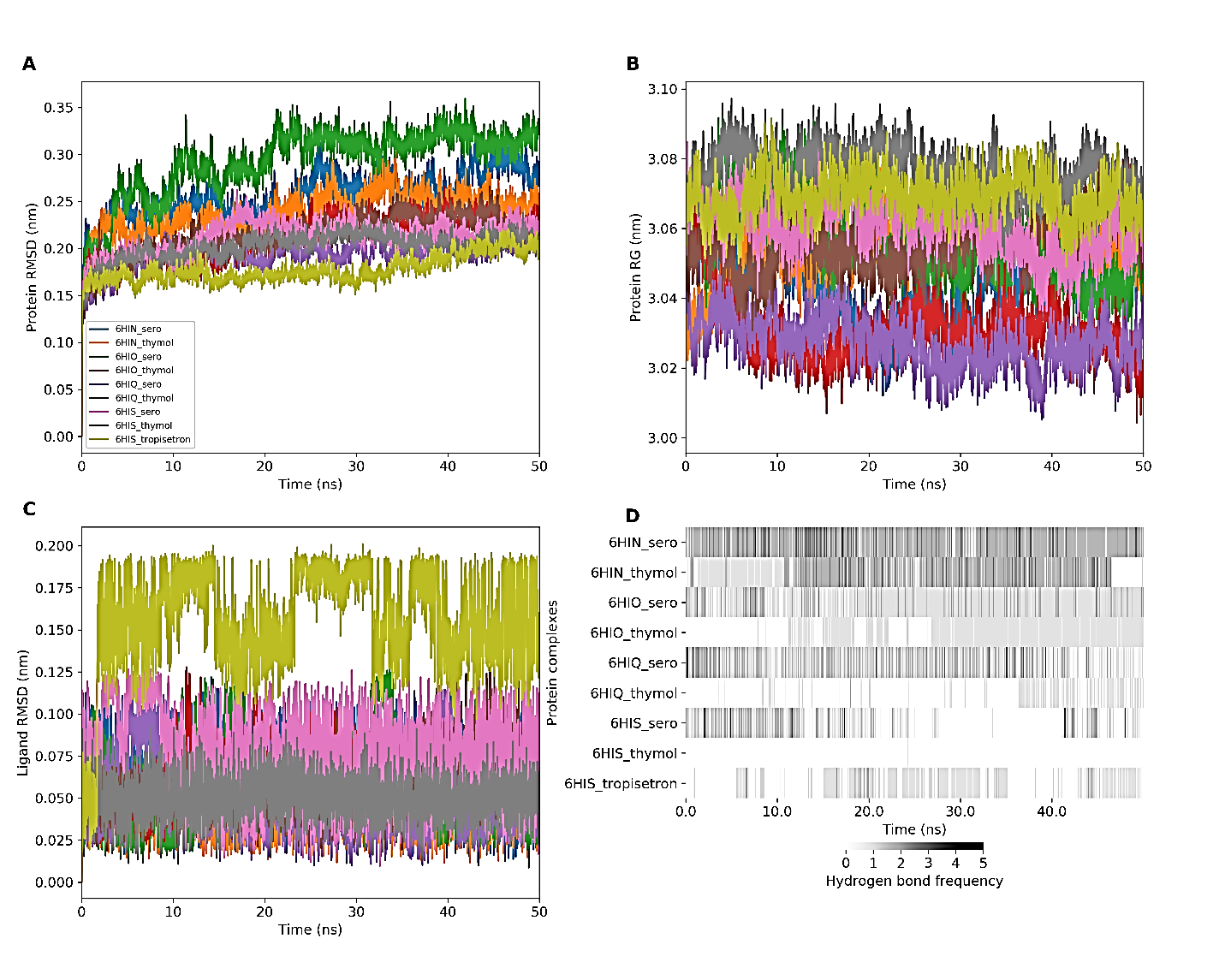
**

**Supporting Figures S9:**

**
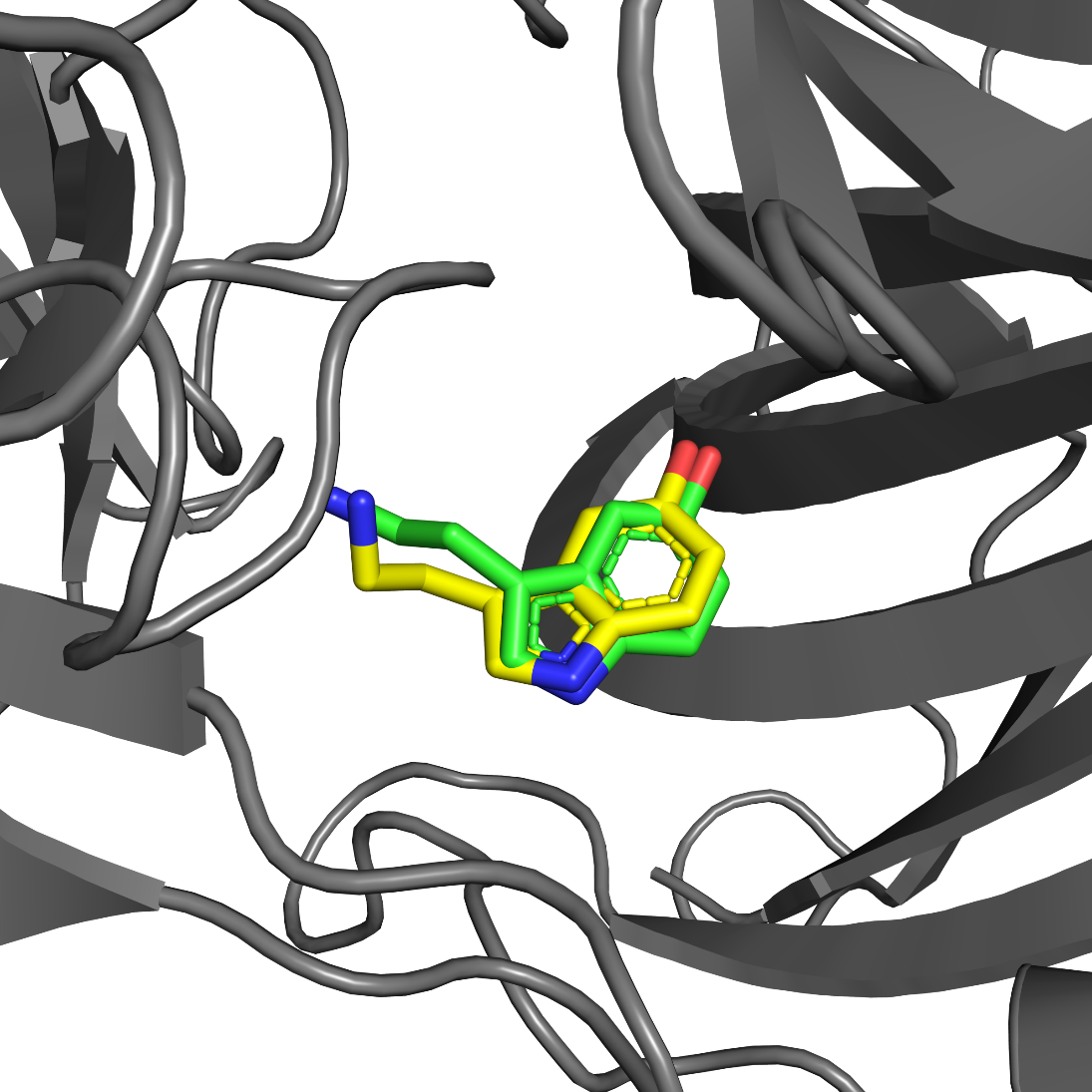
**
